# Evolution of blood biomarker levels following percutaneous atrial septal defect closure in adults

**DOI:** 10.1016/j.ijcha.2020.100582

**Published:** 2020-07-21

**Authors:** Laurie W. Geenen, Lucas Uchoa de Assis, Vivan J.M. Baggen, Jannet A. Eindhoven, Judith A.A.E. Cuypers, Eric Boersma, Jolien W. Roos-Hesselink, Annemien E. van den Bosch

**Affiliations:** Department of Cardiology, Erasmus University Medical Center Rotterdam, the Netherlands

**Keywords:** Atrial septal defect, Blood biomarkers, Percutaneous closure, Adult congenital heart disease

## Abstract

**Background:**

We sought to assess the effects of percutaneous atrial septal defect (ASD) closure on blood biomarker levels that possibly reflect reverse cardiac remodeling. Therefore, this study investigated temporal changes in six blood biomarkers following percutaneous ASD closure in adults.

**Methods:**

In this prospective observational cohort study, adults with ASD type II scheduled for percutaneous closure were included (2012–2016). NT-proBNP, high-sensitive troponin-T (hs-TnT), high-sensitive C-reactive protein (hs-CRP), red blood cell distribution width (RDW), growth differentiation factor-15 (GDF-15) and galectin-3 were measured one day prior to ASD closure and one day, three months and one year post ASD closure, and changes were evaluated using paired T-tests. Echocardiographic measurements were obtained.

**Results:**

Fifty patients were included (median age 50 years, 62% women, 32% NYHA II). At baseline, biomarker levels were elevated in a substantial number of patients; NT-proBNP n = 22 (45%), hs-TnT n = 6 (13%) hs-CRP n = 19 (40%), galectin-3 n = 5 (11%) and GDF n = 10 (23%). One day after ASD closure, significant increases of hs-TnT (median change (Δ) = 12 ng/L), hs-CRP (Δ = 1.9 mg/L), GDF-15(Δ = 129 pg/mL) and RDW (Δ = 0.1%) were observed, and a decrease in galectin-3 (Δ = −1.0 ng/mL). Consequently, 92% had at least one abnormal biomarker directly after closure. At three months biomarker levels returned to baseline, and while echocardiographic measures 1 year post closure were indicative of reverse cardiac remodeling, biomarker levels did not further decrease.

**Conclusion:**

Percutaneous ASD closure in adults leads to a direct increase in most blood biomarkers, in particular hs-CRP and hs-TnT. After three months, biomarkers returned to baseline levels and remained stable up to one year.

## Highlights

• A substantial number of adults with unrepaired ASD have elevated biomarker levels.
• Percutaneous ASD closure in adults leads to acute changes in most biomarker levels.
• TnT, CRP, RDW, GDF-15 increase immediate after ASD closure and galectin-3 decreases.
• Long-term reverse cardiac remodeling was not reflected by a decrease in biomarkers.
• Novel biomarkers can help to elucidate mechanisms of reverse cardiac remodeling.

## 1 Introduction

Atrial septal defect (ASD) accounts for approximately 13% of all congenital heart defects and is the second most prevalent congenital heart defect [1]. Although ASDs are often detected and closed during childhood, it is not uncommon that ASD’s remain undiagnosed and clinically manifest at adult age. Long-term persistence of left-to-right shunts can trigger adverse cardiac remodeling including right ventricular dilatation and dysfunction, and increased right-sided cardiac pressures [2]. In some cases, systemic-to-pulmonary shunts affect the pulmonary vasculature leading to pulmonary arterial hypertension (PAH) [3]. PAH is associated with a substantial decrease in life expectancy [4] and ASD closure is therefore indicated in patients with a hemodynamic relevant ASD [5]. However, some of the patients that undergo ASD closure at adult age, still develop PAH [6].

ASD closure can lead to reverse cardiac remodeling, including improvement in left ventricular (LV) function and decreases in right ventricular dimensions and pressures [7], accompanied by an improvement in exercise capacity [8] and NYHA class [9]. However, in older patients, LV diastolic dysfunction may cause development of acute congestive left heart failure following ASD closure as a result of the sudden flow increase [10]. Better comprehension of the pathophysiological consequences and reverse cardiac remodeling after ASD closure at advanced age, may be helpful to adjust monitoring and determine follow-up strategies of patients after ASD closure.

Blood biomarkers can be of help to gain insight in cardiac remodeling after ASD closure by reflection of certain biological processes, such as inflammation or myocardial injury. N-terminal pro-B natriuretic peptide (NT-proBNP) is released in response to increased cardiac wall stress, troponin-T and C-reactive protein (CRP) are indicative of cardiac myocyte injury and inflammation, respectively. Also novel blood biomarkers such as growth differentiation factor-15 (GDF-15) and galectin-3 have been linked to the pathophysiology and progression of heart failure [11]. While NT-proBNP and troponins have been investigated in the light of ASD closure in adults, very limited data is available on other biomarker responses following ASD closure. This study aimed to investigate the temporal changes of NT-proBNP, high sensitivity troponin-T (hs-TnT), high sensitivity CRP (hs-CRP), red cell distribution width (RDW), galectin-3 and GDF-15 following percutaneous ASD closure in adults.

## 2 Methods

### 2.1 Study design and population

This is a single-center prospective observational cohort study. Adult patients with an ASD type II scheduled for percutaneous ASD closure at our center were enrolled between March 2012 and December 2016. Patients were excluded if they lived abroad, had renal dysfunction (estimated glomerular filtration rate < 30 ml/min/1.73 m^2^), or if patients had switched to a surgical ASD closure procedure. The study protocol was approved by the medical ethical committee and written informed consent was provided by all patients. The study was performed in accordance with the principles outlined in the Declaration of Helsinki.

Baseline was defined as the visit scheduled prior to ASD closure. Physical examination, 12-lead electrocardiography, echocardiography and venous blood sampling were performed. Decision for percutaneous ASD closure was at the discretion of the treating physician following a multi-disciplinary discussion by experts, according to the ESC guidelines [5]. ASD closure was performed using the Amplatzer or Gore Septal device. Hemodynamic measurements were taken during the catheter intervention, prior to defect closure. Physical examination, ECG and venous blood sampling were repeated one day after closure at 3 (range 1–5) months and 12 (range 8–20) months after ASD closure. Two-dimensional trans thoracic echocardiography (TTE) was performed at baseline (before ASD closure) and 12 months after closure using a commercially available ultrasound system (iE33 or Epic, Philips Medical Systems, Best, the Netherlands). Routine echocardiographic indexes including right atrial pressure and right ventricular systolic pressures (RVSP), were measured in accordance with the guidelines [12], [13]. Right atrial and right ventricular measurements were primarily performed in RV-focused views if available, otherwise the four chamber view was used. Echocardiography studies were blinded for biomarker levels.

### 2.2 Biomarker measurements

Blood biomarkers were measured for study purposes only and not used for clinical decision-making. Blood samples were transferred to the clinical chemistry laboratory within 2 hours. NT-proBNP and RDW were measured in fresh blood samples. NT-proBNP was measured using a commercial electrochemiluminescense immunoassay (Roche Diagnostics). The rest of the samples were aliquoted and securely stored at −80 °C in the central Biobank. Hs-TnT, hs-CRP and GDF-15 were measured by batch analyses in thawed serum samples using electrochemiluminescence immunoassays (for hs-TnT and GDF-15) or an immunoturbidimetric assay (for hs-CRP) (Roche Diagnostics, Basel, Switzerland). Lower limits of detection were 3 ng/L for hs-TnT, 0.3 mg/L for hs-CRP and 400 ng/L for GDF-15. Galectin-3 levels were measured using the ARCHITECT chemiluminescent microparticle immunoassay (Abbot Diagnostics, Hoofddorp, the Netherlands) and had a lower limit of quantitation of ≤4.0 ng/mL. The upper limits of normal were defined as >15 pmol/L for NT-proBNP, >14 ng/L for hs-TnT, >3 mg/L for hs-CRP and >16% for RDW. For galectin-3 sex specific reference values were applied; 21.3 ng/mL for women and 16.9 ng/mL for men [14]. For GDF-15 an age-specific cut-off was applied; >920 pg/mL for patients aged <50 years and 1330 pg/mL for patients aged >50 years [15].

### 2.3 Statistical analysis

Continuous data are given as mean ± SD or median and interquartile range [IQR]. Baseline characteristics are shown for all patients, and shown for patients below or above the median age in our study separately. Comparison of clinical characteristics between patients below or above the median age was performed using the unpaired *T*-test or Mann Whitney *U* test for continuous variables. For comparison of categorical variables the Fisher Exact test was used. Biomarker levels were 2log transformed for further analysis because of skewed distributions. Correlation between patient characteristics and baseline blood biomarkers were assessed with Pearson or Spearman correlation coefficient, depending on the distribution of the data. Measurements of TTE, ECG and blood biomarkers between two measurement moments (baseline with 1 day post closure, and baseline with 1 year post closure) were compared using the paired *T*-test if normally distributed, otherwise the related-samples Wilcoxon signed rank test was used. The McNemar test was used to compare paired categorical data between baseline and 1 year post ASD closure. Biomarker levels are presented as boxplots showing median, quartiles and ranges. The median of the differences in biomarker levels in between baseline and one day after ASD closure in each patient is presented as delta (Δ), with the corresponding inter quartile range. A two-sided p-value < 0.05 was considered statistically significant. Data was analyzed using SPSS, Version 24.0, for Windows.

## 3 Results

### 3.1 Baseline characteristics

A total of 68 patients were screened prior to percutaneous ASD closure, of which 50 patients were enrolled in the study. A flowchart of patient selection is shown in Supplementary File 1. Median age was 50 [IQR 38–62] years, 31 patients were women (62%) and 16 patients (32%) patients were NYHA class II (Table 1). Twelve patients (24%) had a history of atrial fibrillation and in 7 of these patients atrial fibrillation was present at baseline ECG. Normal left ventricular systolic function was present in 41 patients (84%). One patient was diagnosed with PAH before ASD closure. Elevated biomarker levels at baseline were present in a substantial proportion of the patients: NT-proBNP in 22 patients (45%), hs-TnT in 6 patients (13%), hs-CRP in 19 patients (40%), galectin-3 in 5 patients (11%), GDF-15 in 10 patients (23%). (Table 1)

**Table 1:** Baseline characteristics of adult patients before percutaneous closure of the atrial septal defect.

	**Complete cases, n (%)**	**All patients (n = 50)**	**Patients < 50 years (n = 25)**	**Patients > 50 years (n = 25)**	**p-value**
**Clinical Characteristics**					
Age at closure, years	50 (100)	50 [38–62]	38 [31–46]	62 [58–68]	–
Sex, women	50 (100)	31 (62)	17 (68)	14 (56)	0.561
Body mass index, kg/m^2^	50 (100)	26.6 ± 4.8	25.7 ± 5.6	27.5 ± 3.7	0.135
Systolic blood pressure, mmHg	50 (100)	138 ± 20	129 ± 16	147 ± 20	<0.001
NYHA class	50 (100)				0.032
I		34 (68)	21 (84)	13 (52)	
II		16 (32)	4 (16)	12 (48)	
Cardiac medication use*	50 (100)	16 (32%)	0 (0)	16 (64%)	<0.001
Systemic hypertension	50 (100)	9 (18)	0 (0)	9 (36)	0.002
Coronary artery disease	50 (100)	2 (4)	0	2	–
History of atrial fibrillation	50 (100)	12 (24)	1 (4)	11 (44)	0.002
**Electrocardiography**					
Rhythm	49 (98)				0.023^#^
Sinus rhythm		41 (84)	24 (96)	17 (71)	
Atrial fibrillation		7 (14)	0 (0)	7 (29)	
Pacemaker rhythm		1 (2)	1 (4)	0	
Heart rate, beats/minute	49 (98)	71 ± 12	71 ± 11	71 ± 14	0.920
PR interval, ms^†^	41 (100)	171 ± 28	163 ± 26	184 ± 27	0.020
QRS duration, ms	49 (98)	112 ± 22	106 ± 20	117 ± 22	0.059
Complete RBBB	49 (98)	10 (20)	2 (8)	8 (32)	
**Echocardiography**					
Normal LV function	49 (98)	42 (84)	25 (100)	17 (68)	0.004
LV end-diastolic dimension, mm	50 (100)	46.7 ± 6.3	45.9 ± 4.8	47.4 ± 7.5	0.466
E/E’	35 (70)	8.3 [6.7–9.2]	8.3 [7.0–9.9]	8.3 [6.4–8.7]	0.882
E/A	35 (70)	1.1 [1.0–1.4]	1.3 [1.1–1.8]	0.88 [0.78–1.1]	<0.001
Left atrial dimension, mm	49 (98)	40.4 ± 9.6	36.3 ± 5.1	44.6 ± 11.4	0.007
Right atrial area, mm	49 (98)	23.3 [18.7–28.4]	22.3 [18.3–25.5]	25.3 [19.3–37.4]	0.075
RVED area, cm^2^	49 (98)	34.9 ± 8.5	33.2 ± 7.2	36.5 ± 9.6	0.242
RVED basal diameter, mm	50 (100)	49.5 ± 6.8	48.6 ± 6.9	50.3 ± 6.8	0.410
RVED apex to base length, mm	49 (98)	85.6 ± 9.2	86.6 ± 8.1	84.6 ± 10.3	0.435
RV fractional area change, %	49 (98)	41.0 ± 9.5	42.5 ± 8.5	39.4 ± 10.4	0.280
TAPSE, mm	46 (92)	27.8 ± 5.7	28.7 ± 4.4	26.8 ± 6.9	0.316
Right atrial pressure	44 (88)				0.126
3 mmHg (range 0–5 mmHg)		36 (72)	21 (84)	15 (60)	
8 mmHg (range 5–10 mmHg)		8 (16)	2 (8)	6 (24)	
RV systolic pressure, mmHg	39 (78)	28 [26–38]	26 [24], [25], [26], [27], [28]	37 [32–44]	<0.001
**Hemodynamics^$^**					
Right atrial pressure, mmHg	41 (82)	7 [6], [7], [8], [9]	7 [5], [6], [7], [8]	8 [6], [7], [8], [9], [10], [11], [12]	0.095
Mean PAP, mmHg	46 (50)	19 [16], [17], [18], [19], [20], [21], [22], [23], [24]	17 [16], [17], [18], [19], [20]	22 [19], [20], [21], [22], [23], [24], [25], [26]	0.007
**Biomarker measurements**					
NT-proBNP, pmol/L	48 (96)	14 [5], [6], [7], [8], [9], [10], [11], [12], [13], [14], [15], [16], [17], [18], [19], [20], [21], [22], [23], [24], [25], [26], [27], [28], [29], [30], [31]	6 [3], [4], [5], [6], [7], [8], [9], [10], [11], [12], [13], [14], [15]	26 [10–92]	<0.001
Hs-TnT, ng/L	48 (96)	5 [3], [4], [5], [6], [7], [8], [9], [10]	3 [3], [4], [5]	7 [5], [6], [7], [8], [9], [10], [11], [12], [13], [14], [15]	<0.001
Hs-CRP, mg/L	48 (96)	2.0 [0.9–4.2]	1.2 [0.5–3.7]	2.4 [1.3–5.3]	0.032
RDW, %	48 (96)	12.7 [12.1–13.2]	12.5 [11.9–13.0]	12.9 [12.3–13.4]	0.098
GDF-15, pg/mL	43 (86)	749 [546–1288]	404 [547–622]	1123 [776–1486]	<0.001
Galectin-3, ng/mL	45 (90)	14.2 [11.7–16.2]	13.3 [14.5–14.3]	14.9 [12.3–17.2]	0.082

**Table legend:** Values are given in mean ± SD, median [IQR] or n (%). *use of ACE-inhibitor (n = 3), angiotensin receptor blockers (n = 4), beta blocker (n = 13) or diuretics (n = 7). ^#^compares sinus rhythm versus any other rhythm ^†^Atrial fibrillation and pacemaker rhythms are not included. ^$^Measured during percutaneous ASD closure procedure.

**Abbreviations:** NYHA = New York Heart Association, RBBB = right bundle branch block, LV = left ventricular, RV = right ventricular, RVED = right ventricular end-diastolic, TAPSE = tricuspid annular plane systolic excursion, PAP = pulmonary artery pressure, NT-proBNP = N-terminal pro-B natriuretic peptide, hs-TnT = high sensitivity troponin-T, hs-CRP = high sensitivity C-reactive protein, RDW = red cell distribution width, GDF-15 = Growth differentiation factor-15

Adults above 50 years of age more frequently used cardiac medication, more often had atrial fibrillation and an abnormal left ventricular function, larger left atrial dimensions and higher mean pulmonary artery pressures (mPAP). NT-proBNP, hs-TnT, hs-CRP and GDF-15 levels before closure were significantly higher in patients aged >50 years (Table 1).

### 3.2 Biomarkers and associations with baseline characteristics

Higher NT-proBNP levels were associated with higher NYHA class. Higher levels of NT-proBNP, hs-TnT, RDW and GDF-15 were associated with the presence of atrial fibrillation. Hs-TnT was the only biomarker that showed a significant correlation with QRS duration and PR interval. Most biomarker levels at baseline correlated with the left atrial dimension, right atrial area and RVSP. NT-proBNP, hs-TnT and RDW positively correlated with invasively measured right atrial pressures and mPAP (Table 2).

**Table 2:** Correlations between biomarker levels and baseline clinical characteristics as well as ASD closure related characteristics.

	**NT-proBNP**	**hs-TnT**	**Hs-CRP**	**RDW**	**GDF-15**	**Gal-3**
	*r*	*r*	*r*	*r*	*r*	*r*
**Clinical Characteristics**						
Age at closure	0.51^***^	0.74^***^	0.25	0.18	0.75^***^	0.30*
Women	0.16	−0.55^***^	0.07	0.10	−0.09	−0.09
Body mass index	0.14	0.213	0.26	0.37^**^	0.12	0.29
Systolic blood pressure	0.09	0.45^**^	0.11	−0.30*	0.41^**^	−0.09
NYHA class	0.37*	0.25	0.21	−0.03	0.31*	−0.12
Cardiac medication use	0.52^***^	0.63^***^	0.36*	0.27	0.57^***^	0.47^**^
Hypertension	0.36*	0.48^**^	0.25	0.20	0.41^**^	0.06
Atrial fibrillation	0.44^**^	0.54^***^	0.19	0.40^**^	0.40^**^	0.26
**Electrocardiography**						
Heart rate	−0.03	0.00	0.06	−0.11	0.01	−0.27
PR interval †	0.07	0.40*	0.11	0.07	0.28	0.29
QRS duration	0.01	0.39^**^	0.01	0.03	0.25	0.21
**Echocardiography**						
LV end-diastolic dimension	0.05	0.17	−0.19	0.19	0.09	0.01
E/E’	0.08	0.13	0.41*	−0.04	0.09	−0.23
E/A	0.12	−043*	0.00	0.817	−0.35	0.03
Left atrial dimension	0.54^***^	0.48^**^	0.15	0.41^**^	0.47^**^	0.38^**^
Right atrial area	0.45^**^	0.35*	0.01	0.31*	0.25	0.18
RVED area	0.20	0.27	0.05	0.28	0.16	0.30*
RVED basal diameter	−0.03	0.09	0.15	−0.01	−0.01	0.08
RVED apex to base length	−0.26	0.08	−0.24	−0.15	−0.011	−0.010
RV fractional area change	−0.20	−0.19	0.19	−0.00	−0.21	0.05
TAPSE	−0.34*	−0.18	0.14	−0.10	−0.17	0.00
Right atrial pressure of 8 mmHg	0.47^**^	0.29	0.09	0.34*	0.30	0.18
RV systolic pressure, mmHg	0.55^***^	0.47^**^	0.37*	0.21	0.47**	0.29
**Hemodynamics**						
Right atrial pressure	0.26	−0.05	−0.04	0.43^**^	0.16	0.15
Mean PAP	0.49^**^	0.37*	0.27	0.37*	0.21	0.29
**Procedural characteristics**						
Diameter of ASD, balloon	0.27	0.04	0.26	0.31	−0.01	0.23
Diameter of ASD, echo	0.03	−0.02	−0.25	0.08	−0.02	0.04
Device size	0.05	0.07	0.11	−0.16	0.14	0.07
Device size, bsa indexed	0.08	−0.05	0.13	−0.19	0.14	0.14

**Table legend:** Significant correlations are printed in bold with the corresponding level of significance shown by the number of asterisks (* p-value < 0.05, ^**^p-value < 0.01, ^***^ p-value < 0.001).

**Abbreviations:** NYHA = New York Heart Association, RBBB = right bundle branch block, LV = left ventricular, RV = right ventricular, RVED = right ventricular end-diastolic, TAPSE = tricuspid annular plane systolic excursion, PAP = pulmonary artery pressure, NT-proBNP = N-terminal pro-B natriuretic peptide, hs-TnT = high sensitivity troponin-T, hs-CRP = high sensitivity C-reactive protein, RDW = red cell distribution width, GDF-15 = growth differentiation factor-15, gal-3 = galectin-3.

NT-proBNP correlated with all biomarkers except hs-CRP and of all mutual biomarker correlations, the strongest correlation was observed between NT-proBNP and GDF-15 (r = 0.74, p < 0.001) (Supplementary File 2).

### 3.3 ASD closure and acute effects on biomarker levels

Percutaneous ASD closure was performed using the Amplatzer device (n = 38) or the Gore Septal occluder (n = 12), with a median device size of 24 [IQR 18–28] mm. An elevated mPAP (≥25 mmHg) was measured in 11 patients (23%) prior to ASD closure. Eight (16%) patients had a minimal rest shunt after device implantation. One patient underwent device removal due to cardiac tamponade; this patient was excluded from further analysis. No further complications occurred.

**Fig. 1:**
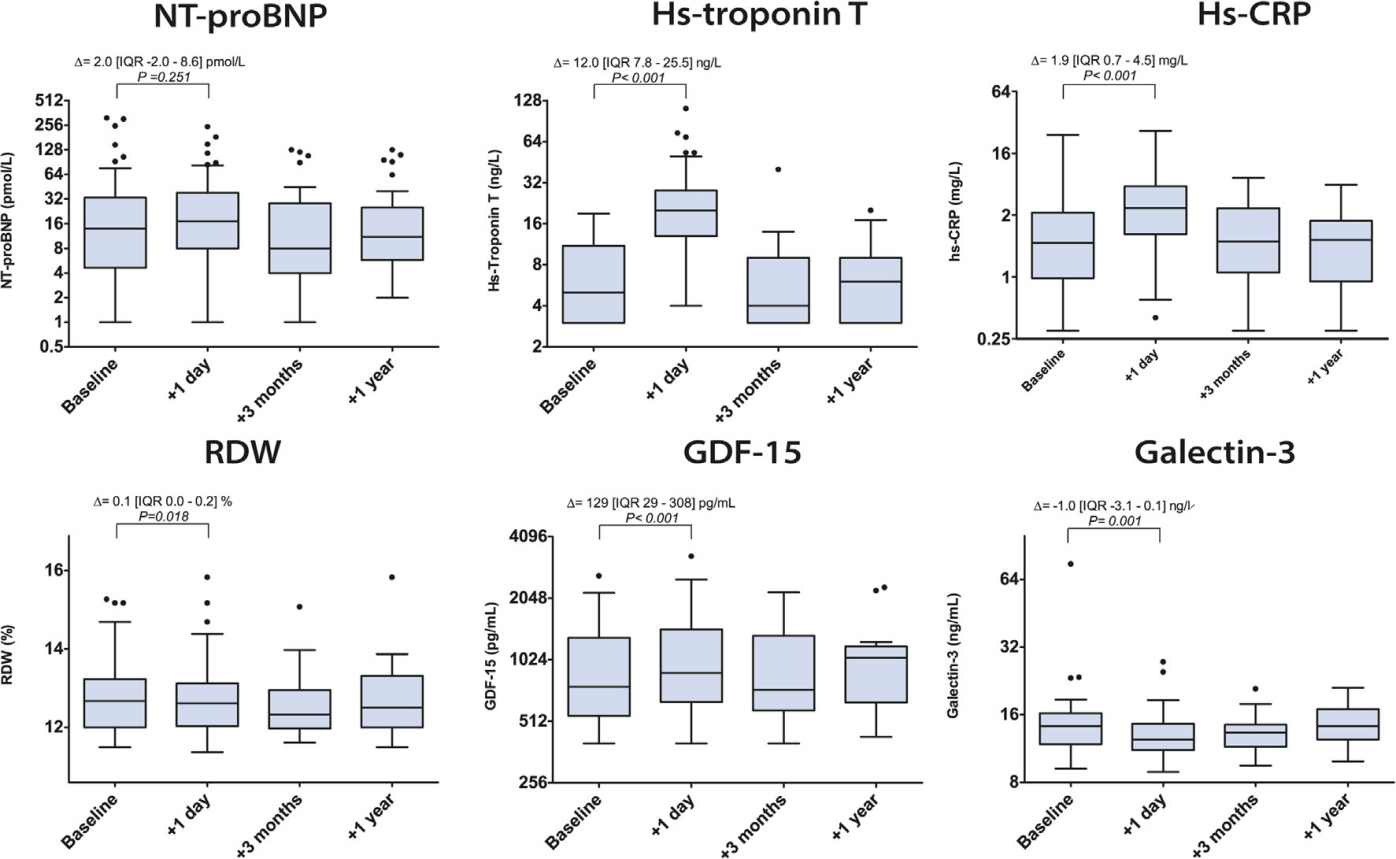
Serial biomarker levels before closure (baseline) and 1 day, 3 months and 1 year after percutaneous ASD closure in adults. X-axis are on the 2-log scale. Delta (Δ) represents the median of the difference in the biomarker level before and one day ASD after closure in. P-value represents level of significance of the paired *t*-test. **Abbreviations**: NT-proBNP = N-terminal pro B type natriuretic peptide, GDF-15 = growth differentiation factor 15, RDW = red cell distribution width, hs-CRP = high sensitive c-reactive protein. (For interpretation of the references to colour in this figure legend, the reader is referred to the web version of this article.)

**Fig. 2:**
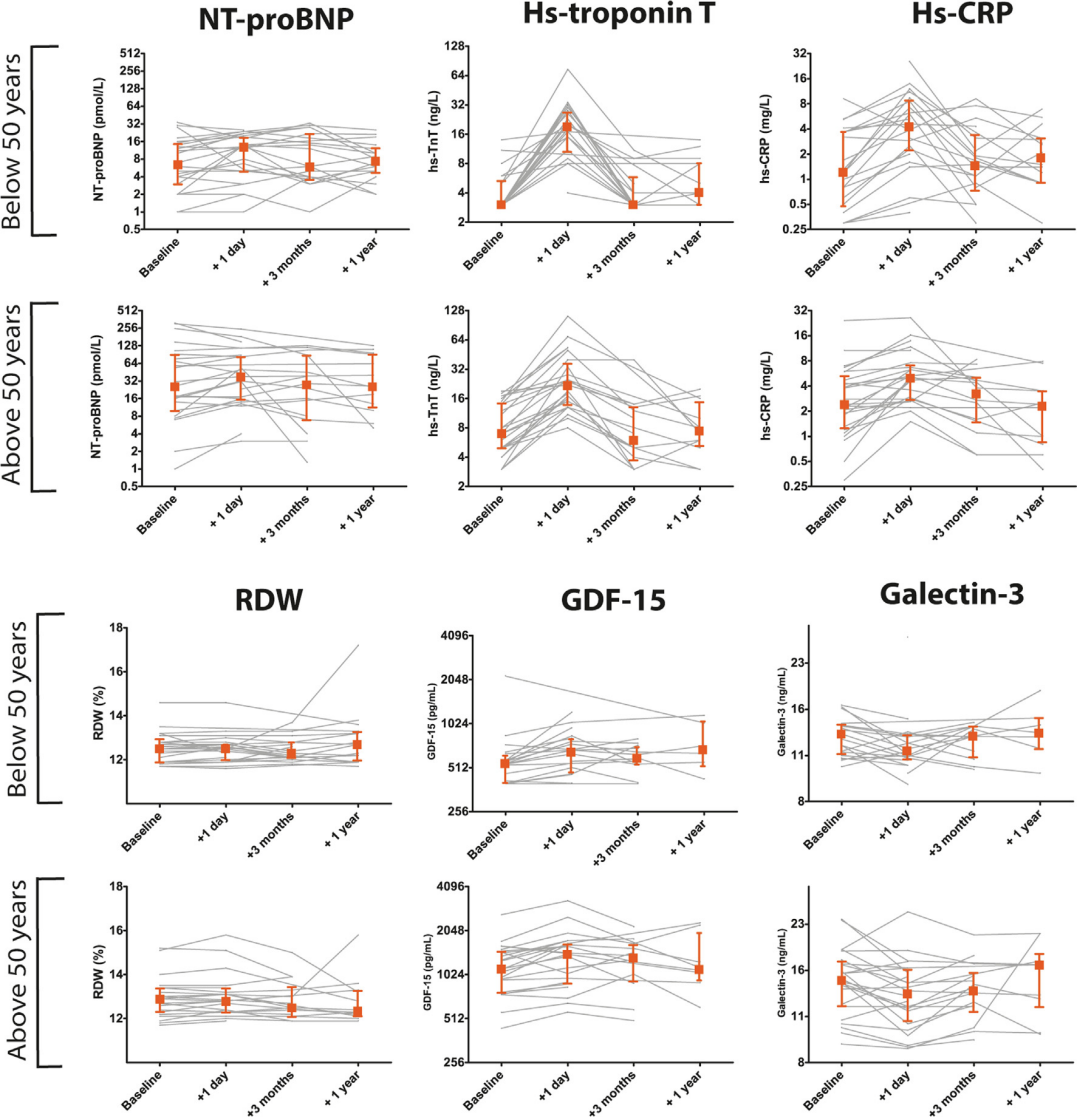
Individual biomarker trajectories of NT-proBNP, hs-troponin T, hs-CRP, RDW, GDF-15 and galectin-3 stratified according to age at ASD closure. gray lines represent individual biomarker trajectories over time. Square represents the median biomarker level, together with the 25th and 75th percentile (indicated by the error bars). **Abbreviations**: NT-proBNP = N-terminal pro B type natriuretic peptide, GDF-15 = growth differentiation factor 15, RDW = red cell distribution width, hs-CRP = high sensitive c-reactive protein. (For interpretation of the references to colour in this figure legend, the reader is referred to the web version of this article.)

One day post ASD closure a significant increase in concentrations of hs-TnT, GDF-15, RDW, hs-CRP and a decrease of galectin-3 was observed (Fig. 1 and Supplementary File 3). Of note, NT-proBNP was elevated in 26 patients (52%) 1 day post closure, but no significant increase was observed relative to baseline levels. Increases were most pronounced in hs-TnT and hs-CRP; one day after closure 35 (73%) and 30 (63%) patients had an elevated hs-TnT or hs-CRP, respectively. In only 4 patients (8%) none of the biomarkers was elevated one day post closure. No significant differences were found in the acute absolute biomarker changes (levels one day post ASD closure minus baseline levels) between patients aged < 50 years or > 50 years (Fig. 2).

The acute increase in hs-TnT and hs-CRP showed no significant correlation with mPAP or right atrial pressures before closure and with device disk sizes also after indexing the disk size for BSA. Also no significant differences were observed in the acute biomarker changes and the type of the device used (data not shown).

### 3.4 Long-term effects of ASD closure

Three months up to one year post ASD closure blood biomarker levels returned to initial values and thereafter remained stable and no further decreases were observed. An elevated NT-proBNP was still present in 12 patients (40%) 1 year after ASD closure, while hs-TnT was elevated in only 3 patients (11%) and hs-CRP in 8 patients (30%). Considering echocardiographic changes 1 year after ASD closure, dimensions of the right ventricle were significantly decreased as well as the tricuspid annular plane systolic excursion, while the left ventricular end-diastolic dimension had increased. (Table 3) No differences in baseline characteristics were observed in patients with and without blood biomarker measurements one year post ASD closure, though it should be kept in mind that biomarker measurements were missing in some of the patients at 1 year (Supplementary Files 4&5).

**Table 3:** Echocardiographic and electrocardiographic measurements before percutaneous atrial septal defect closure in adults (baseline) and 1 year after ASD closure.

	**Baseline (prior to ASD closure)**		**1 year post ASD closure**		**p-value**
	Complete cases, n(%)	Mean ± SD or median [IQR]	Complete cases, n(%)^#^	Mean ± SD or median [IQR]	
**Clinical characteristics**					
NYHA class	50 (100)		42 (86)		0.020
class I		34 (68)		34 (81)	
class II		16 (32)		8 (19)	
**Electrocardiography**					
PR-interval*	41 (100)	171 ± 28	30 (75)	159 ± 31	0.001
QRS duration	49 (98)	112 ± 22	38 (78)	109 ± 21	0.110
**Echocardiography**					
LV end-diastolic dimension, mm	50 (100)	46.7 ± 6.3	41 (84)	49.1 ± 4.9	0.009
E/E'	35 (70)	8.3 [6.7–9.2]	31 (63)	8.5 [7.3–10.4]	0.808
E/A	35 (70)	1.1 [1.0–1.4]	33 (67)	1.05 [0.8–1.5]	0.683
Left atrial dimension, mm	49 (98)	40.4 ± 9.6	40 (82)	38.3 ± 10.6	0.084
Right atrial area, cm^2^	49 (98)	23.3 [18.7–28.4]	40 (82)	18.0 [14.5–22.5]	<0.001
RVED area, cm^2^	49 (98)	34.9 ± 8.5	40 (82)	28.2 ± 7.5	<0.001
RVED basal diameter, mm	50 (100)	49.5 ± 6.8	42 (86)	44.0 ± 6.8	<0.001
RVED apex to base length, mm	49 (98)	85.6 ± 9.2	40 (82)	82.1 ± 9.3	0.001
RV fractional area change, %	49 (98)	41.0 ± 9.5	40 (82)	37.7 ± 7.8	0.155
TAPSE, mm	46 (92)	27.8 ± 5.7	29 (59)	23.2 ± 3.6	<0.001
Right atrial pressure	44 (88)		35 (71)		0.096
Range 0–5 mmHg		36 (72)		32 (91)	
Range 5–10 mmHg		8 (16)		6 (9)	
RV systolic pressure, mmHg	39 (78)	28 [26–38]	29 (59)	27 [23–36 (4]	0.024

**Table legend:**^#^One patient was excluded from the 1-year analysis because of cardiac device erosion. *Atrial fibrillation, atrial flutter or pacemaker rhythm are not included.

**Abbreviations**: ASD = atrial septal defect, NYHA = New York Heart Association, LV = left ventricular, RVED = right ventricular end-diastolic, TAPSE = tricuspid annular plane systolic excursion, RV = right ventricular.

As post-hoc sensitivity analysis, we repeated the analysis of the acute and long-term effects of ASD closure on biomarkers levels excluding patients who did not have a 1-year measurement of the biomarker of interest. The results did not differ from the main results.

## 4 Discussion

This study investigated temporal changes in the blood biomarkers NT-proBNP, hs-TnT, hs-CRP, RDW, galectin-3 and GDF-15, following percutaneous ASD closure in adults. Percutaneous ASD closure in adults was associated with an acute increase in most blood biomarkers, in particular hs-TnT and hs-CRP, and resulted in at least one elevated biomarker level in 92% of the patients. These findings indicate that hemodynamic changes occurring after ASD closure in adults are likely accompanied by an inflammatory response, cardiomyocyte damage and several pathways of cardiac remodeling. Three months post ASD closure biomarker levels returned to baseline levels and remained stable up to one year after ASD closure. While older patients more often had LV dysfunction and higher right-sided pressures as well as higher biomarker levels before ASD closure, no distinct differences were observed regarding the course of the biomarkers over time in these patients.

### 4.1 Acute effects of ASD closure

The acute increase in hs-TnT following after ASD closure likely reflects cardiomyocyte injury. Two possible explanations can be given; 1) the device insertion and catheter procedure provoke myocardial damage or 2) the acute LV volume overload due to shunt cessation post ASD closure induces myocardial injury. Of note, diagnostic catheterizations itself have not been associated with short-term troponin increases [16], [17]. The increase in hs-TnT is in line with Tárnok et al., who found a short-term cardiac troponin I increases after ASD closure in both children as well as adults [17]. Along with hs-TnT, a pronounced increase in hs-CRP one day post closure was observed, suggesting an inflammatory response following ASD closure. A similar short-term hs-CRP increase has been observed in patients undergoing transradial or transfemoral diagnostic catheterization [18], yet chronic inflammation expressed by elevated hs-CRP has also been proposed to play a role in the pathophysiology of adult congenital heart disease (ACHD) [19]. Whether the increase in hs-CRP is due to shunt termination or attributable to the catheter intervention itself, or a combination of both, is therefore questionable. Nevertheless, it indicates that ASD closure has a significant direct effect on biomarker levels in adults and that elevated levels of hs-TnT and hs-CRP are present in the majority of patients.

Galectin-3 was the only biomarker that decreased in response to ASD closure. This biomarker is known to induce fibroblast proliferation and ventricular dysfunction [20] and inhibition of galectin-3 has shown to terminate further progression of adverse cardiac remodeling in rats [21]. Given the direct galectin-3 decrease after ASD closure, the shunt cessation must have induced a relief in cardiac burden at some point. In a similar manner, levels of GDF-15 increased following ASD closure. GDF-15 is known to protect ventricular cardiomyocytes and induce hypertrophic growth in these cells [22]. Hence, it seems that cardiac protective and adaptive mechanisms are put into operation directly after shunt cessation. To the best of our knowledge, galectin-3, GDF-15 and RDW levels have not been investigated in the context of ASD closure in adults, while all three biomarkers have been associated with the prognosis in ACHD [14], [23], [24].

In our study no significant increase in NT-proBNP was found one day after ASD closure, this is in contrast to several studies that did find an increase [25], [26]. An explanation for the absence of an increase in our study may be the presence of already relatively high levels of NT-proBNP before closure. This may be due to a larger proportion of older patients in our study with co-existence of more cardiovascular comorbidities such as hypertension and atrial fibrillation. The diminished left ventricular function in the patients aged over 50 years, may also have contributed to increased NT-proBNP levels. Patients aged below 50 years old at closure had significantly lower NT-proBNP levels before closure; however, no significant increase in NT-proBNP was observed directly after closure.

### 4.2 Long-term effects of ASD closure

Our study did not observe a decrease in biomarker levels between baseline and 3 months to 1 year post ASD closure. There may be several explanations for the absent decrease in biomarker levels on the long-term. Firstly, reverse cardiac remodeling following ASD closure may have reached a plateau 3 months after closure. Secondly, cardiac remodeling may not be entirely reversible and the molecular or cellular adaptation may have ended after a certain period of time. Thirdly, long-term cardiac remodeling may not be reflected by the biomarkers measured in our study. In addition to these explanations, missing biomarker levels at 3 and 12 months may have prevented us to observe a decrease, as missing biomarker measurements in our study may have more likely been present in in asymptomatic patients in who lower biomarker levels can be expected. However, no differences were observed in baseline characteristics between patients with and without biomarker levels 1 year post ASD closure.

The direct increase in biomarkers following ASD closure are likely to indicate cardiac adaptation at a molecular or cellular level, however cardiac remodeling after ASD closure visible by echocardiography is considered to take a longer period of time. The significant improvement in NYHA class and decrease in right-heart dimensions as well as right-sided pressures suggests presence of long-term cardiac remodeling after ASD closure in our study. These results are in line with a previous study in 23 patients, who all underwent closure at 50 years or older [9]. Differences in right ventricular end-diastolic dimensions were measured 6 weeks and 1-year after ASD closure, and a significant decrease over time was found. However, similarly to our study, the authors did not find a significant change in BNP in the long-term [9]. In contrast, another study did report a decrease in NT-proBNP 6 and 12 months after ASD closure in adults, which was associated with decreases in RVSP and right ventricular end-diastolic volume [27].

The decrease in TAPSE observed post ASD closure is in line with previous studies [7], [28], [29], and could most likely be explained by recovering of the right ventricular geometry as a result of the reduced right ventricular volume overload after ASD closure. Following the Frank-Starling law, a reduction in volume leads to more efficient pumping of the right ventricle at a lower functional state.

### 4.3 Clinical implications

Findings of this study suggest that ASD closure has an immediate effect on a molecular and cellular level and this change may anticipate perceptible long-term echocardiographic changes in cardiac remodeling. ASD closure in adults leads to acute profound changes in biomarkers, and presence of elevated biomarkers levels post ASD closure seem to be a common reaction. This should be taken into account when measuring biomarkers directly after closure for the interpretation of abnormal levels. The findings of this study warrants further in-depth research whether these biological pathways could be a potential therapeutic target to prevent adverse cardiac remodeling, or enhance reverse cardiac remodeling after closure.

Biomarkers play a role in the prognosis of patients with pulmonary hypertension [30], [31], though it is not clear what role biomarkers play in the development of PAH in adults with an ASD. Whether the biomarker response after ASD closure will be predictive of long-term echocardiographic changes or development of PAH and thus can contribute to the follow-up strategy of these patients, is a highly relevant question that should be further investigated. Based on this study, novel biomarkers like GDF-15 and galectin-3 can, apart from more commonly known biomarkers, contribute to the understanding and future research in this field.

The prognostic value of NT-proBNP, hs-TnT, hs-CRP, GDF-15, RDW and galectin-3 has previously been determined in ACHD patients [14], [19], [23], [24]. Based on biomarker levels found in these studies, the ASD patients in our study had relatively high levels and should, based on the observed biomarker levels, have a far less favorable prognosis than is generally the case in ASD patients [32]. The prognostic value of these biomarkers is therefore likely to be different in ASD patients compared to other ACHD patients and it could be worthwhile to investigate their long-term prognostic value. It would be of great interest to investigate whether biomarker responses following ASD closure, can help to determine the follow-up frequency in patients after ASD closure, which should be addressed in future studies.

### 4.4 Limitations

Because of the number of missing biomarker measurements, deviation in time of blood sampling visits, and the relatively small number of patients, caution should be taken when interpreting the results of this study. The sample size of this study is small, limiting the power to perform statistical analyses, though compared to previous studies on biomarker levels following ASD closure, this can be considered a relatively large study. Differences between two related measurements were investigated using appropriate statistical testing (i.e. paired tests). However these tests do not take into account regression towards the mean; a statistical phenomenon introduced by random measurement variability, that describes the effect of extreme values becoming less extreme when repeatedly measured based on chance. Regression towards the mean may have biased our results, most likely to an underestimation of the changes in biomarker levels [33].

As this is an observational study on patient-level, this study cannot confirm any causal relation between biomarker release and cardiac remodeling in adults who undergo ASD closure. This should be kept in mind when interpreting the results of our study.

This study relied on patient blood-sampling for biomarker measurements but logistical or patient-related issues resulted in some patients not having biomarker measurements at certain follow-up moments, in particular 3 months and 1-year follow-up measurements. The missing biomarker measurements were randomly determined by logistical or patient-related issues, but not death. Our observations considering the biomarker trajectories from 3 months to 1-year may still have been biased, in particular the missing data may have prevented us to observe a decrease in biomarker levels on the long-term. Nevertheless, our study was able to show distinct increases in biomarker levels directly after ASD closure.

## 5 Conclusion

A substantial number of adults with an unrepaired ASD have elevated blood biomarker levels, even in absence of pulmonary hypertension. Percutaneous ASD closure leads to a direct increase in hs-TnT, hs-CRP, RDW and GDF-15, and a decrease in galectin-3. This suggests that ASD closure is followed by a comprehensive cardiac response including inflammation, cardiomyocyte injury and other pathophysiologic processes. Three months post ASD closure, blood biomarker levels return to baseline levels and remain stable up to one year. While cardiac remodeling was clearly reflected by echocardiographic changes at 1 year of follow-up, this was not reflected by a distinct decrease in blood biomarker levels on the long-term.

## CRediT authorship contribution statement

**Laurie W. Geenen:** Investigation, Conceptualization, Formal analysis, Writing - original draft. **Lucas Uchoa Assis:** Investigation, Formal analysis, Writing - review & editing. **Vivan J.M. Baggen:** Investigation, Methodology, Resources, Writing - review & editing. **Jannet A. Eindhoven:** Conceptualization, Methodology, Resources, Writing - review & editing. **J.A.A.E. Cuypers:** Resources, Writing - review & editing. **Eric Boersma:** Conceptualization, Methodology, Writing - review & editing. **Jolien W. Roos-Hesselink:** Conceptualization, Resources, Funding acquisition, Writing - review & editing. **Annemien E. den Bosch:** Conceptualization, Resources, Supervision, Funding acquisition, Writing - review & editing.
