# Supplementary material for: Evolution of blood biomarker levels following percutaneous atrial septal defect closure in adults
Source: Int J Cardiol Heart Vasc. 2020 Jul 21;30:100582. doi: 10.1016/j.ijcha.2020.100582 (PMC7378682; doi:10.1016/j.ijcha.2020.100582)
Supplement: Supplementary data 1 [file mmc1.docx]

**Evolution of biomarker levels following percutaneous atrial septal defect closure in adults**

Laurie W. Geenen, Lucas Uchoa de Assis, Vivan J.M. Baggen, Jannet A. Eindhoven, Judith A.A.E. Cuypers, Eric Boersma, Jolien W. Roos-Hesselink, Annemien E. van den Bosch

**Supplementary Files**

**List of files page**

**Supplementary File 1**. Flowchart of the patient selection process...……………………….……………..1

**Supplementary File 2**. Mutual biomarker correlations……………………………………………………2

**Supplementary File 3**. Individual biomarker trajectories of NT-proBNP, hs-troponin T, hs-CRP, RDW, GDF-15 and galectin-3 following percutaneous ASD closure in adults....………………….……………...3

**Supplementary File 4**. Baseline characteristics according to patients with and without biomarker measurements 1 year post ASD closure…………………………………………………………………….4

**Supplementary File 5.** Number of biomarker measurements at each specific follow-up moment………..6

**
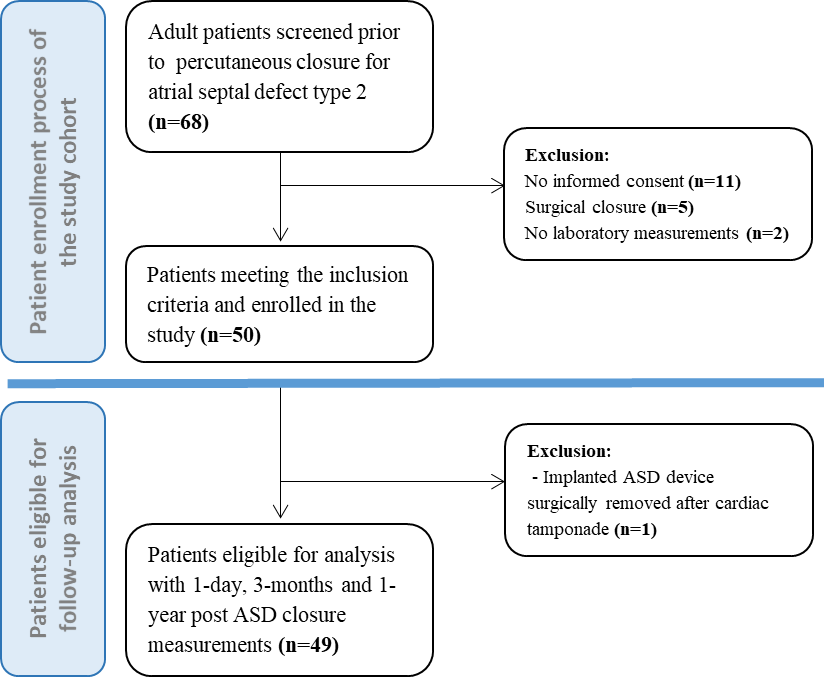
**

**Supplementary File 1.** Flowchart of the patient selection process. Both the patient enrollment process of the study cohort and the subsequent number of patients eligible for analysis with follow-up measurements are shown.

**Abbreviations:** ASD= atrial septal defect

|  | **Hs-TnT** | **Hs-CRP** | **RDW** | **GDF-15** | **Galectin-3** |
| --- | --- | --- | --- | --- | --- |
|  | *r* | *r* | *r* | *r* | *r* |
| **NT-proBNP** | 0.38^**^ | 0.27 | 0.53^***^ | 0.74^***^ | 0.45^**^ |
| **hs-TnT** |  | 0.15 | 0.22 | 0.69^***^ | 0.28 |
| **hs-CRP** |  |  | 0.16 | 0.15 | 0.05 |
| **RDW** |  |  |  | 0.34^*^ | 0.31^*^ |
| **GDF-15** |  |  |  |  | 0.24 |
| **Galectin-3** |  |  |  |  |  |

**Supplementary File 2.** Mutual biomarker correlations at baseline **Table legend:** **^*^** p-value < 0.05, **^**^**p-value < 0.01, **^***^** p-value <0.001
**Abbreviations**: NT-proBNP= N-terminal pro-B natriuretic peptide, hs-TnT= high sensitive troponin-T, hs-CRP= high sensitive C-reactive protein, RDW= red cell distribution width, GDF-15= Growth differentiation factor-15.


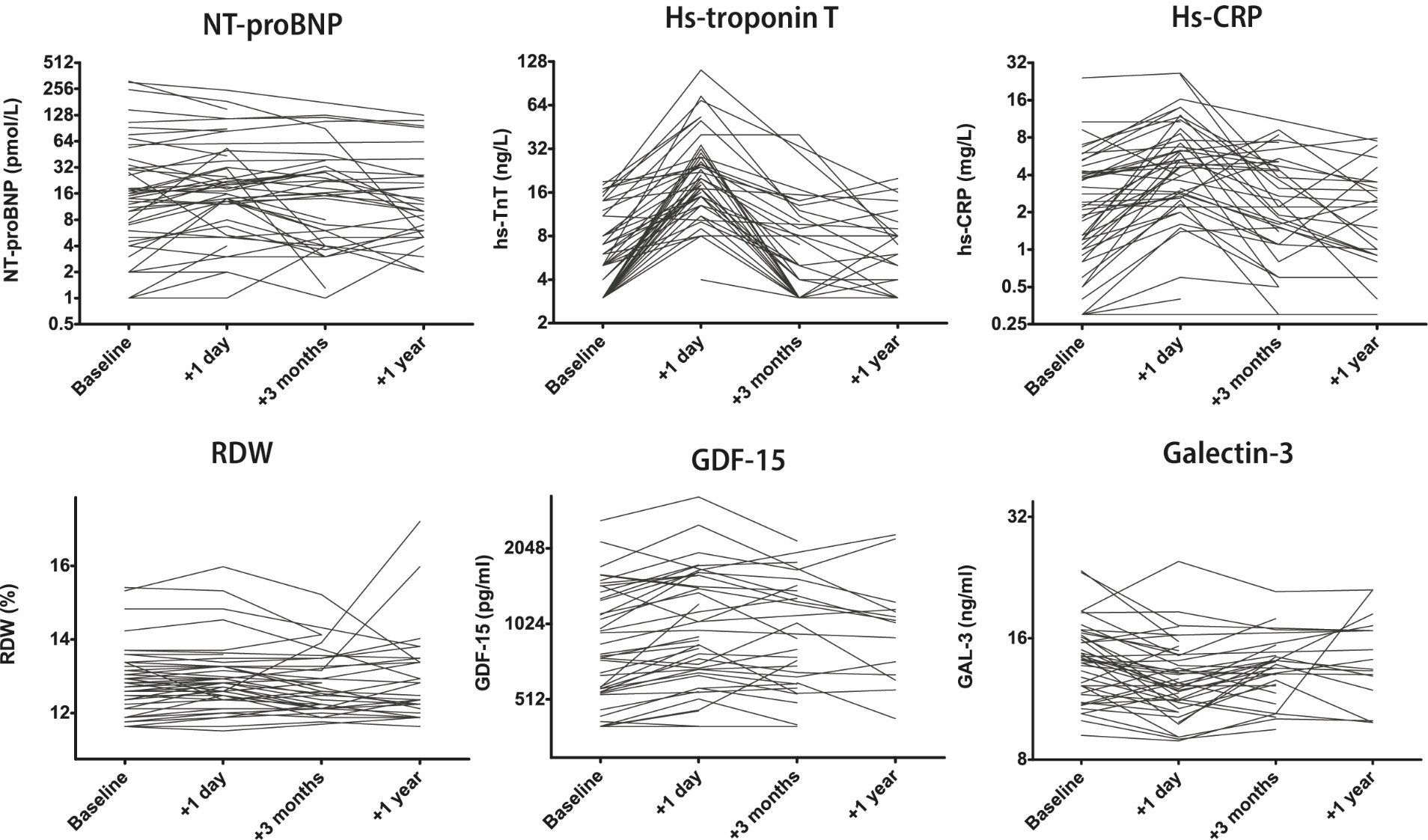


**Supplementary File 3.** Individual biomarker trajectories in adults following percutaneous ASD closure.
X-axis is on the 2-log scale. Biomarker measurements of each patient at each follow-up moment is connected by a line, representing the biomarker evolution over time.
**Abbreviations**: NT-proBNP= N-terminal pro B type natriuretic peptide, GDF-15= growth differentiation factor 15, RDW= red cell distribution width, hs-CRP= high sensitive c-reactive protei

|  | **1-year biomarker measurement** | |  |
| --- | --- | --- | --- |
|  | **Absent** | **Present** | **p-value** |
| No. of patients | 19 | 31 |  |
| **Clinical characteristics** |  |  |  |
| Age at closure, years | 51 [36-62] | 48 [38-65] | 0.960 |
| Women | 14 (74) | 17 (55) | 0.237 |
| BMI, kg/m^2^ | 26.7 ± 6.3 | 26.6 ± 3.7 | 0.952 |
| Systolic blood pressure, mmHg | 144 ± 22 | 134 ± 19 | 0.103 |
| NYHA class II | 7 (37) | 9 (29) | 0.756 |
| Cardiac medication use^*^ | 6 (32) | 10 (32) | 1.00 |
| Systemic hypertension | 4 (21) | 5 (16) | 0.715 |
| Coronary artery disease | 0 (0) | 2 (7) | 0.485 |
| History of atrial fibrillation | 4 (21) | 8 (26) | 1.00 |
| **Electrocardiography** |  |  |  |
| Sinus rhythm | 18 (95) | 23 (74) | 0.128 |
| Heart rate, beats/minute | 69 ± 10 | 73 ± 14 | 0.797 |
| PR interval, ms^†^ | 178 ± 38 | 169 ± 27 | 0.654 |
| QRS duration, ms | 116 ± 25 | 109 ± 19 | 0.406 |
| Complete RBBB | 10 (53) | 10 (32) | 0.033 |
| **Echocardiography** |  |  |  |
| Normal LV function | 18 (95) | 24 (77) | 0.224 |
| LV end diastolic dimension, mm | 44.3 ± 5.8 | 48.1 ± 6.2 | 0.080 |
| E/E’ | 7.1 [6.4-8.7] | 8.5 [7.0-11.6] | 0.198 |
| E/A | 1.06 [0.87-1.33] | 1.20 [0.99-1.58] | 0.353 |
| Left atrial dimension, mm | 37.6 ± 9.2 | 42.0 ± 9.7 | 0.188 |
| Right atrial area, mm | 23.3 [17.7-25.8] | 23.7 [19.2-28.8] | 0.407 |
| RVED area, cm^2^ | 32.4 ± 5.6 | 36.2 ± 9.6 | 0.147 |
| RVED basal diameter, mm | 48.2 ± 5.4 | 50.2 ± 7.5 | 0.347 |
| RVED apex to base length, mm | 83.6 ± 7.4 | 86.8 ± 10.0 | 0.335 |
| RV fractional area change, % | 43 ± 10 | 40 ± 9 | 0.320 |
| TAPSE, mm | 28.2 ± 5.9 | 27.5 ± 5.7 | 0.813 |
| RV systolic pressure, mmHg | 30 [26-44] | 28 [25-37] | 0.323 |
| **Hemodynamics^$^** |  |  |  |
| Right atrial pressure | 8 [5-10] | 7 [6-9] | 0.924 |
| Mean PAP | 18 [15-25] | 19 [17-24] | 0.712 |
| **Biomarker measurements** |  |  |  |
| NT-proBNP, pmol/L | 12 [3-44] | 14 [6-31] | 0.457 |
| hs-TnT, ng/L | 4 [3-8] | 5 [3-12] | 0.360 |
| hs-CRP, mg/L | 1.7 [1.0-4.1] | 2.3 [0.8-4.3] | 0.657 |
| RDW, % | 12.7 [11.9-13.2] | 12.7 [12.1-13.3] | 0.579 |
| GDF-15, pg/mL | 761 [560-1367] | 748 [543-1284] | 0.848 |
| Galectin-3, ng/mL | 14.6 [11.6-16.7] | 13.9 [11.7-15.9] | 0.337 |

**Supplementary File 4.** Baseline characteristics stratified according to patients without biomarker levels 1 year post ASD closure and patients with biomarker measurements 1 year post ASD closure.
**Table legend:** Values are given in mean ± SD, median [IQR] or n(%). P-value is given for the comparison between patients with and without biomarker levels. Mann Whitney U test was used for comparison of continuous variables and the Fisher exact test for categorical variables.
^*^use of ACE-inhibitor (n=3),angiotensin receptor blockers (n=4), beta blocker (n=13) or diuretics (n=7). ^#^compares sinus rhythm against any other rhythm ^†^Atrial fibrillation and pacemaker rhythms are not included. ^$^Measured during percutaneous ASD closure procedure.
**Abbreviations**: BMI= body mass index, NYHA= New York Heart Association, RBBB= right bundle branch block, LV= left ventricular, RV= right ventricular, RVED= right ventricular end-diastolic, TAPSE = tricuspid annular plane systolic excursion, PAP= pulmonary artery pressure

|  | **Number of available biomarker measurements** | | | |
| --- | --- | --- | --- | --- |
| ***n=50 patients*** | Prior to ASD closure (baseline) | 1 day post ASD closure | 3 months post ASD closure | 1 year post ASD closure |
| NT-proBNP | 49 (98) | 48 (96) | 33 (67) | 30 (61) |
| Hs-TnT | 48 (96) | 48 (96) | 31 (63) | 27 (55) |
| Hs-CRP | 48 (96) | 48 (96) | 31 (63) | 27 (55) |
| RDW | 49 (98) | 45 (90) | 32 (65) | 28 (57) |
| GDF-15 | 43 (86) | 44 (86) | 25 (51) | 14 (29) |
| Galectin-3 | 45 (90) | 45 (90) | 26 (53) | 17 (35) |
|  |  |  |  |  |

**Supplementary File 5.** Number and % of biomarker measurements for each specific biomarker at each specific endpoint in adults who underwent percutaneous ASD closure.  **Abbreviations:** ASD= atrial septal defect, NT-proBNP= N-terminal pro B type natriuretic peptide, hs-TnT= high sensitivity troponin T, hs-CRP= high sensitivity C-reactive protein, RDW= red cel distribution width, GDF-15= growth differentiation factor 15
